# Fearful Faces do Not Lead to Faster Attentional Deployment in Individuals with Elevated Psychopathic Traits

**DOI:** 10.1007/s10862-017-9614-x

**Published:** 2017-06-30

**Authors:** Sylco S. Hoppenbrouwers, Jaap Munneke, Karen A. Kooiman, Bethany Little, Craig S. Neumann, Jan Theeuwes

**Affiliations:** 1 Department of Clinical Psychology, Erasmus University Rotterdam, Burgemeester Oudlaan 50, 3062PA Rotterdam, The Netherlands; 2 Aysel Sabuncu Brain Research Center, Bilkent University, Ankara, Turkey; 3 Department of Psychology, Bilkent University, Ankara, Turkey; 4 Department of Experimental and Applied Psychology, Vrije Universiteit Amsterdam, Amsterdam, Netherlands; 5 Department of Psychology, University of North-Texas, Denton, TX USA

**Keywords:** Psychopathy, Attention, Top-down attention, Response modulation hypothesis, Fear

## Abstract

In the current study, a gaze-cueing experiment (similar to Dawel et al. 2015) was conducted in which the predictivity of a gaze-cue was manipulated (non-predictive vs highly predictive). This was done to assess the degree to which individuals with elevated psychopathic traits can use contextual information (i.e., the predictivity of the cue). Psychopathic traits were measured with the Self-Report Psychopathy Scale-Short Form (SRP-SF) in a mixed sample (undergraduate students and community members). Results showed no group difference in reaction times between high and non-predictive cueing blocks, suggesting that individuals with elevated psychopathic traits can indeed use contextual information when it is relevant. In addition, we observed that fearful facial expressions did not lead to a change in reaction times in individuals with elevated psychopathic traits, whereas individuals with low psychopathic traits showed speeded responses when confronted with a fearful face, compared to a neutral face. This suggests that fearful faces do not lead to faster attentional deployment in individuals with elevated psychopathic traits.

## Introduction

Psychopathy is a severe personality disorder that encompasses two interrelated factors (i.e., an interpersonal-affective and a behavioral-antisocial lifestyle factor (Hare and Neumann 2006; Paulhus et al. 2016)). The first factor represents personality features such as grandiosity, a charming and manipulative interpersonal style, a lack of empathy and a lack of emotional depth. The second factor represents the unstable lifestyle that is characteristic of psychopathy, including features such as impulsivity, recklessness and versatile criminal behavior. Various theories aim to explain affective and cognitive abnormalities that are associated with psychopathy. The response modulation theory of psychopathy proposes that the emotional and behavioral deficits of psychopathy are not pan-situational but are rather situation-specific. Depending on the situation, top-down goals that a psychopathic individual sets, determine which information will be processed (Newman and Baskin-Sommers 2011). As such, the response modulation theory attributes a large role to attention in explaining psychopathic behavior.

Recently, Dawel and colleagues (Dawel et al. 2015) conducted an experiment to assess the evidence for different theories of psychopathy: the ‘distress-specific’ hypothesis, the ‘attention-to-the-eyes’ hypothesis and the ‘enhanced-selective-attention’ hypothesis were investigated.The distress-specific hypothesis is derived from Blair’s violence inhibition mechanism and, in short, states that psychopathic individuals are insensitive to (signs of) distress in others (Blair 2013). The amygdala-based ‘attention-to-the-eyes’ hypothesis (Dadds et al. 2006) postulates that psychopathic individuals have an amygdala deficit which leads them to deploy little attention to the eyes. As the eyes convey important information regarding the emotional state of another person, psychopathic individuals do not process emotional information, in particular fearful facial expressions.

Dawel et al. (2015) used a gaze-cueing experiment with emotional faces, in which the direction of the eye-gaze served as an attentional cue that was non-predictive for where the target would appear. While overall accuracy and reaction times did not differ between groups with high or low callous-unemotional (CU) traits, the high CU-group appeared to use the cue less and less as the experiment progressed. The results were taken to be in line with the response modulation theory as the high CU-group suppressed the goal-irrelevant cue. However, the Dawel et al. study was missing one critical experimental condition: To make the claim that psychopathy is related to the suppression of *irrelevant* information it is important to show that psychopathic individuals do use this information when it is *relevant*.

In the current study, we aimed to extend the findings of Dawel et al. (2015) and determine whether individuals with elevated psychopathic traits can use such information when it is relevant. To this end, we included a condition in which the gaze-cue was highly predictive for where the target would appear (i.e., 80% validity). We hypothesized that individuals with elevated psychopathic traits would not show a difference between the predictive (valid cues on 80% of the trials) and non-predictive (valid cues on 50% of the trials) blocks. This would indicate that elevated psychopathic traits preclude the processing of secondary information, independent of the relevance of this information.

## Methods

### Participants

We tested a mixed community sample (*N* = 88) consisting of 74 students of the Vrije Universiteit Amsterdam and 14 non-students recruited from the Amsterdam community (14 males, mean age = 24.3, SD = 5.9; four participants did not provide age information; age range: 18–35). All participants had normal or corrected-to-normal vision and reported no history of mental illness. Prior to the start of the experiment, informed consent was obtained from all individual participants included in the study. The student participants were recruited through the University’s online participant pool system. Of these students, 24 were undergraduate Psychology students who participated for course credits, and 50 were students from a range of undergraduate and post-graduate courses who participated for money. Non-student community members were recruited through flyers which were distributed around Amsterdam and online advertisements on classified advertisements websites and social media websites.

### Materials and Design

Psychopathy: Participants started the experimental session by filling out the Self-Reported Psychopathy – Short Form questionnaire (SRP-SF; Paulhus et al. 2016) aimed at providing quantifiable scores for the different factors associated with psychopathic traits. The SRP is strongly positively correlated with the PCL-R (Neumann and Pardini 2014; Williams et al. 2007), the Youth Psychopathic Traits Inventory (Neumann and Pardini 2012), and a psychopathy self-report based on the five-factor model of personality (Lynam et al. 2011; Miller et al. 2013). The questionnaire consisted of 29 statements to which the participants had to indicate to what extent they agreed or disagreed with the statement on a Likert-scale ranging from 1 (strongly disagree) to 5 (strongly agree). Statements included sentences such as: “Most people are wimps” and “I like to see fist-fights”. The SRP-SF provides a score for each participant on four independent facets associated with psychopathy (similar to the SRP-III; (Newman and Declercg 2009) and the Psychopathy Checklist-Revised (PCL-R; Hare, 2003)): 1. Deficits in interpersonal behavior such as “glibness” or being manipulative (7 items), 2. Deficits in affective behavior such as lack of remorse, callousness and being unemotional (7 items), 3. Deviant lifestyle behavior such as being overly impulsive or being prone to boredom (7 items), 4. Antisocial behavior such as criminal behavior and general behavioral control problems (8 items). These four facets can be recombined into two general factors (Factor 1 and Factor 2), the first factor relating to deficits in interpersonal (Facet 1) and affective behavior (Facet 2), whereas the second factor (Facet 3 and Facet 4) is associated with antisocial behavior in its broadest form. See Table 1 for further information on psychopathy scores.

**Table 1:** Demographic information showing the mean, standard deviation and range of the SRP-SF scores cores per group for both SRP-SF factors and the SRP-SF total score. SRP-SF F1 = SRP-SF Factor 1; SRP-SF F2 = SRP-SF Factor 2

	Mean ± standard deviation (SD)		t-value	Cohen’s d	Range	
	Low group	Elevated group			Low group	Elevated group
SRP-SF total	43 ± 3.9 (*n* = 31)	70 ± 8.6 (*n* = 24)	15.59	−4.04	33–49	61–87
SRP-SF F1	19 ± 2.7 (*n* = 30)	35 ± 5.4 (*n* = 23)	14.545	−3.85	14–23	29–55
SRP-SF F2	23 ± 2.0 (*n* = 25)	38 ± 4.9 (*n* = 13)	14.243	−4.22	19–27	33–47

While we recruited 88 participants, we created a low and elevated psychopathic traits group by selecting participants. As per the guidelines in the manual of the Self-Reported Psychopathy – Short Form questionnaire (SRP-SF; Paulhus et al. 2016), the elevated psychopathic traits group scored 60 or higher (*N* = 24) on the SRP-SF Total score and the low psychopathy group scored 50 or below (*N* = 31). Eight subjects had an SRP-SF Total score above 70 indicating the presence of extreme psychopathic traits. For SRP-SF Factor 1 of the low psychopathy group scored 23 or below (*N* = 35) and of the elevated psychopathic traits group scored 29 or higher (*N* = 30). For SRP-SF Factor 2 the low psychopathy group scored 26 or below (*N* = 32) and the elevated psychopathic traits group scored 33 or higher (*N* = 15). In total, 74 participants were included in at least one of the low or elevated psychopathy groups. Please see Table 1 for information on the averages and range of the SRP-SF scores.

### Cueing Tasks

After having filled out the questionnaire, participants were placed in a dimly lit and sound attenuated cubicle, where they took part in two separate cueing tasks that were presented on a 22-in. monitor. Viewing distance was kept constant at 75 cm by using a chin rest. Matlab 2015a & Psychtoolbox 3 (Brainard, 1997; Pelli, 1997) were utilized to code and present the experiment.

The cueing tasks were similar to those used by Dawel et al. (2015). Participants were presented with an arrow or gaze-cue that could indicate the location of an upcoming target to which participants were instructed to respond as fast as possible. Compared to Dawel et al. (2015), a number of critical changes were introduced. Two factors were manipulated in a blocked manner. First, cues could either be highly predictive, i.e., they predicted the target location correctly on 80% of the trials (high predictive blocks), whereas in a separate block the cue was non-predictive of the target location (50% valid; non-predictive blocks). Prior to the start of each trial, the participants were explicitly informed about the predictive nature of the cue. In addition, the cue could consist of a simple arrow pointing left or right (arrow-cueing blocks) or a face with eyes gazing left or the right as an indication as to where the target would appear (gaze-cueing blocks). We used the same face images Dawel et al. used (from the Radboud Faces Database; (Langner et al. 2010). The gaze-cue could either consist of a neutral face or a fearful face (the happy face condition found in the study by Dawel et al. was left out), which were mixed within blocks. Block predictivity and cue-type (arrow or gaze) were combined to create four different block types (gaze cue - non-predictive, gaze cue - high predictive, arrow cue - non-predictive & arrow cue - high predictive), of which the order of presentation was counterbalanced over participants. Participants completed all blocks of one cue type (e.g. arrow cues) before conducting the blocks containing the alternate cue type. Each new block type would start by presenting the participants with instructions explaining the particular block type with an emphasis on the predictability of the cue, followed by 20 practice trials to get (re-) acquainted with the task.

Different conditions constituted a different number of blocks and trials due to the different block predictability levels and the use of multiple emotions in the gaze-cueing blocks as opposed to one type of arrow-cue. For the gaze-cueing blocks, the experiment included five high predictive blocks and two non-predictive blocks. This was done to ensure that there were enough invalid trials in the high predictive condition. For the arrow cueing blocks, two high predictive blocks and one non-predictive block were present in the experiment, each block consisting of 80 trials. The total session time lasted approximately two hours (including filling out the questionnaire). In either block type (gaze-cueing, arrow-cueing), high predictive blocks consisted of 64 valid cues and 16 invalid cues, whereas non-predictive blocks consisted of 40 trials of each cue validity. The number of trials containing a fearful facial expression was identical to the number of trials containing a neutral facial expression.

**Fig. 1:**
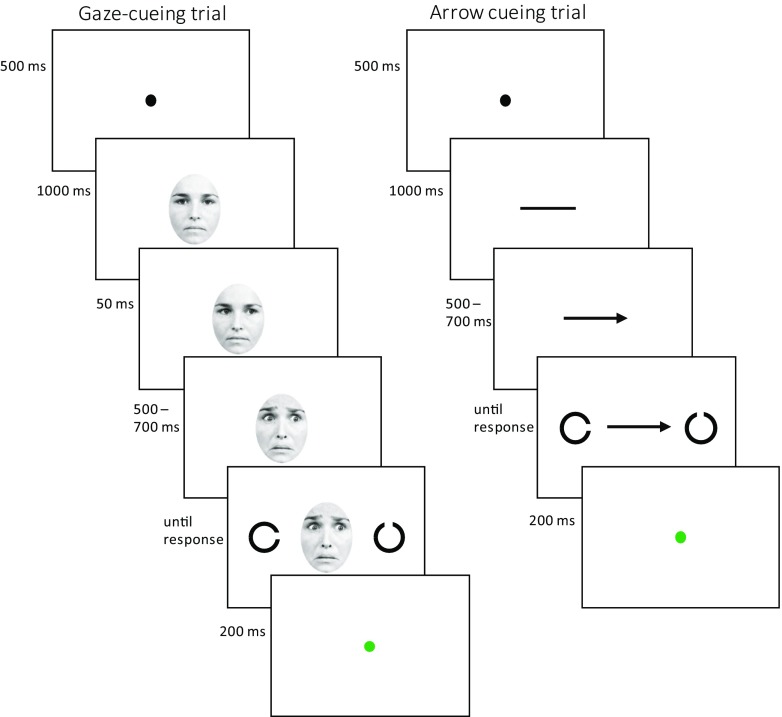
Typical time courses of the two trial types, using gaze- (*left*) or arrow-cues (*right*)

The exact time course of a typical gaze- and arrow-cueing trial can be observed in Fig. 1. Each trial started with a brief fixation dot (500 ms), followed by the presentation of a non-directional neutral face or the (non-directional) shaft of the arrow-cue (1000 ms). All faces consisted of a visual angle of 6.8^0^ by 9.2^0^, whereas the arrow shaft subtended 4.1^0^. In the gaze-cueing task, the neutral non-directional face was briefly replaced by a neutral directional face gazing either to the left or the right (50 ms), before the face would change showing a fearful expression or stay neutral (500, 600 or 700 ms). On arrow-cue trials, an arrow point was added to one side of the shaft, resulting in a directional endogenous cue (500, 600 or 700 ms). While the cue remained on the screen, two Landolt-C figures (diameter 1.7^0^) appeared on the left and the right side of the cue, one Landolt-C bearing a gap on the left or the right side (the non-target), whereas the other stimulus had a gap on the top or the bottom (the target). Participants were instructed to respond to the location of the target opening as fast as possible while the stimuli remained on screen until a response was given. Responses were made on a standard keyboard, by pressing the ‘y’-key for a target opening on top of the stimulus and the ‘b’ key for an opening at the bottom of the stimulus. Feedback was provided immediately after the response in the form of a green (correct) or red (incorrect) fixation dot. Feedback lasted 200 ms after which the next trial was initiated.

### Data Analysis

A mixed design was used to study the relation between psychopathic traits and performance on the cueing tasks. The SRP-SF Total Score, Factor 1 and Factor 2 were used as between-subjects factors in separate ANOVAs, whereas the different experimental conditions were used as within-subjects variables. Within-subjects variables consisted of: ‘block predictivity’ (high predictive or non-predictive), ‘cue validity’ (valid or invalid), and in the gaze-cueing task, ‘emotion’ (neutral or fear). Analyses for the gaze-cueing trials and the arrow-cueing trials were analyzed in separate ANOVAs. Only correct trials were included into the analyses.

The general analyses regarding the task manipulations are reported first, after which the interactions with psychopathy are reported. In all analyses concerning psychopathy, the degree of psychopathy was entered as a between-subjects variable. To explore whether SRP Factor 1 or Factor 2 are more relevant to the use of contextual information or gaze-cueing, separate ANOVAs will be conducted for these factors.

## Results

### Participants

Data from one participant were removed from the data set as performance in one of the conditions was at chance level. Analyses were conducted on the remaining 87 participants.

### Arrow-Cueing: Experimental Manipulation

Table 2 shows the averages and standard deviations of reaction times for all conditions in the arrow- and gaze-cueing blocks. For the arrow-cue trials, the repeated measures GLM with block predictivity (high predictive, non-predictive) and cue validity (valid, invalid) yielded a significant main effect of cue validity, *F*(1,86) = 159.569, *p* < .001, *η*_p_^2^ = .650 indicating participants were faster on valid trials (515 ms) than on invalid trials (607 ms). In addition, a block predictivity by cue validity interaction was observed, *F*(1,86) = 121.724, *p* < .001, *η*_p_^2^ = .586, showing larger cueing effects (the difference in RT between a valid and an invalidly cued target) in the high predictive blocks (Δ136 ms) compared to the non-predictive blocks (Δ49 ms). Not surprising, participants rely more on the arrow cues when they are predictive of the location of the target. Further evidence of an increased use of the arrow-cue in predictive blocks, compared to non-predictive blocks could be observed in an increased speeding up for valid trials in the high predictive, compared to the non-predictive blocks (Δ39 ms; *t*(86) = 7.367, *p* < .001). In addition, a slowed response for invalid cues in the high, compared to the non-predictivity blocks (Δ-49 ms; *t*(86) = 8.163, *p* < .001) was observed.

**Table 2:** Reaction times and standard deviation (in ms) for each condition in the experiment separately for the low- and elevated psychopathy groups

			Gaze-cueing task		Arrow cueing task
			Fear	Neutral	
Low psychopathy group (SRP-SF _Tot_)	High predictive block	Valid	512 (74)	526 (75)	495 (66)
		Invalid	617 (85)	610 (80)	628 (93)
	Low predictive block	Valid	524 (82)	551 (88)	538 (80)
		Invalid	569 (79)	573 (88)	591 (93)
Elevated psychopathy group (SRP-SF _Tot_)	High predictive block	Valid	516 (84)	520 (79)	503 (78)
		Invalid	618 (101)	616 (95)	634 (109)
	Low predicitive block	Valid	557 (91)	562 (88)	540 (94)
		Invalid	587 (90)	593 (89)	587 (105)

### Interaction between Arrow-Cueing and Psychopathy

No interactions between the factors of the arrow-cueing experiment (block predictivity, cue validity) and degree of psychopathic traits were observed (SRP-SF Total: all *p*’s > 0.455; SRP-SF Factor 1 (F1): all *p*’s > 0. 283; SRP-SF Factor 2 (F2): all *p*’s > 0.245). Table 2 shows the reaction times and standard deviations for all conditions in the experiment separately for each group as defined by SRP Total.

### Gaze-Cueing: Experimental Manipulation

A repeated measures GLM with block predictivity (high predictive, non-predictive), emotion (fear, neutral) and cue validity (valid, invalid) yielded two main effects and three interaction effects. A main effect of cue validity was found, *F*(1,86) = 151.754, *p* < .001, *η*_p_^2^ = .638, which indicated that participants were faster on valid trials (533 ms), compared to invalid trials (596 ms). A main effect of emotion was found, *F*(1,86) = 9.672, *p* = .003, *η*_p_^2^ = .101, which showed that participants responded faster when a fearful face was shown (561 ms) than when a neutral face was shown (567 ms).

An interaction effect of block predictivity by cue validity was found, *F*(1,86) = 78.634, *p* < .001, *η*_p_^2^ = .478. Similar to the arrow cueing experiment, this interaction showed a larger cueing effect in the high predictive blocks (Δ97 ms), compared to the non-predictive blocks (Δ29 ms), suggesting that participants used the gaze cues more efficiently when they were predictive than non-predictive of where the target would appear. Additional post-hoc tests showed that the cueing effects were significant in the predictive blocks, *F*(1,86) = 146,929, *p* < .001, *η*_p_^2^ = .631, as well as in the non-predictive blocks, *F*(1,86) = 48.924, *p* < .001, *η*_p_^2^ = .363. The interaction between block predictivity and cue validity shows that the overall predictivity of the cues severely impacts how the cue is used.

An interaction effect of block predictivity *by* emotion was found (at trend level), *F*(1,86) = 3.470, *p* = .066, *η*_p_^2^ = .039. In the high predictive block, the average reaction time for fearful faces was 564 ms and 567 ms for neutral faces. In the non-predictive block, the average reaction time was 559 ms for fearful faces and 568 ms for neutral faces, suggesting that the emotional expression plays a more important role in guiding attention when the cues were non-predictive. However, the difference in response times between neutral and fearful faces was only marginally significant in the high predictive blocks, *F*(1,86) = 3.238, *p* = .075, *η*_p_^2^ = .036, and significant in the non-predictivity blocks, *F*(1,86) = 8.888, *p* = .004, *η*_p_^2^ = .094.

An interaction effect of emotion by cue validity was found, *F*(1,86) = 8.440, *p* = .005, *η*_p_^2^ = .089, showing a larger difference in reaction times between neutral and fearful faces when the target was validly cued (Δ10 ms), compared to when the target was invalidly cued (Δ2 ms). Post-hoc testing showed that the difference in reaction time between neutral and fearful faces was only significant for valid cues, *F*(1,86) = 19.398, *p* < .001, *η*_p_^2^ = .184, but not for invalid cues (F(1,86) = .486, *p* = .497. From a different perspective, the interaction between emotion and cue validity shows a larger cueing effect for fearful faces (Δ67 ms) compared to neutral faces (Δ59 ms). However, post-hoc tests show that both emotions elicit significant cueing effects (fearful: *F*(1,86) = 149.636, *p* < .001, *η*_p_^2^ = .635; neutral: *F*(1,86) = 133.308, *p* < .001, *η*_p_^2^ = .608).

### Interaction between Gaze-Cueing and Psychopathy

Three repeated measures GLMs with block predictivity (high predictive, non-predictive), emotion (fear, neutral) and cue validity (valid, invalid) were conducted in which degree of psychopathy (SRP Total, SRP Factor 1 and SRP Factor 2; low psychopathy group, elevated psychopathy group) was entered as a between-subjects variable. Below, the significant interactions with degree of psychopathy are detailed. Table 2 shows the average reaction times and standard deviations for each condition in the experiment, separately for each group defined by SRP Total.

### SRP Total

A significant interaction effect of block predictivity by SRP total was observed, *F*(1,53) = 4.197, *p* = .045, *η*_p_^2^ = .073. A second GLM for the separate groups showed that the block predictivity effect was marginally significant for the low psychopathy group (*p* = 0.072), and not significant for the elevated psychopathy group (high scoring group; *p* = 0.289). Numerically, participants in the low psychopathy group showed 18 ms faster responses to validly cued targets in high predictive blocks compared to non-predictive blocks. However, they were 43 ms slower on invalid trials in the high predictive blocks compared to the non-predictive blocks. The slowing down for invalid trials was larger than the speeding up on valid trials for participants in the low psychopathy group, which explains the marginally significant block predictivity effect for this group of participants. The main effect of block predictivity for the elevated psychopathy group was not significant, suggesting that the slowing down on invalid trials is equal to the speeding up on valid trials.

A significant interaction effect of cue validity by emotion by SRP total was observed *F*(1,53) = 8.923, *p* = .004, *η*_p_^2^ = .144. Post-hoc comparisons confirmed that the interaction between cue validity and emotion is significant for the low psychopathy group *F*(1,30) = 28.517, *p* < .001, *η*_p_^2^ = .487, but not for the elevated psychopathy group, *F*(1,23) = .146, *p* = .706. This observation indicates that the elevated psychopathy group shows cueing effects of equal magnitude for either emotion, whereas the low scoring group shows a difference in the magnitude of the cueing effect between the two emotions. Post-hoc t-tests confirmed that the difference between the valid fear trial and valid neutral trial is significantly different in the low psychopathy group, *t*(30) = −5.96, *p* < .001 but not in the high psychopathy group, *t*(23) = −1.043, *p* = .308. Table 2 shows that, on valid trials, the low psychopathy group was faster than the elevated psychopathy group when a fearful face was presented. For accuracy, please see (Table 3).

**Table 3:** Accuracy of the low and elevated SRP-SF groups in the emotional gaze-cueing paradigm. Note that in all conditions both groups perform at ceiling level

	High predictive block				Low predictive block			
	Fear valid	Neutral valid	Fear invalid	Neutral invalid	Fear valid	Neutral valid	Fear invalid	Neutral invalid
Low SRP-SF	97.7 (93–100%)	95.8 (85–100%)	97.7 (91–100%)	96.1 (78–100%)	96.5 (85–100%)	95.6 (84–100%)	97.6 (88–100%)	96.5 (83–100%)
High SRP-SF	97.1 (93–99%)	93.0 (73–100%)	97.2 (89–100%)	92.3 (76–100%)	96.1 (76–100%)	94.1 (68–100%)	95.3 (76–100%)	96.0 (84–100%)

### SRP Factor 1

One significant interaction effect of cue validity by emotion by SRP F1 was observed, *F*(1,62) = 8.340, *p* = .005, *η*_p_^2^ = .116. This interaction is similar to the one observed for SRP Total. While the low psychopathy group becomes faster on valid trials when a fearful face is presented, individuals with elevated psychopathic traits are not faster on valid trials when a fearful face is presented, compared to when a neutral face is presented.

### SRP Factor 2

One significant interaction of cue validity by emotion by SRP-SF F2 was observed, *F*(2,45) = 4.476, *p* = .04, *η*_p_^2^ = .09. This interaction is similar to the one observed for SRP Total, While the low psychopathy group becomes faster on valid trials when a fearful face is presented, individuals with elevated psychopathic traits are not faster on valid trials when a fearful face is presented, compared to when a neutral face is presented.

## Discussion

The current study aimed to extend the findings by Dawel et al. (2015). Based on earlier observations (Hoppenbrouwers et al. 2016a; Hoppenbrouwers et al. 2015), the question was raised whether individuals with elevated psychopathic traits are superior at suppressing irrelevant information, or whether they have difficulty integrating contextual information, independent of the relevance of this information. To this end, we used two cueing experiments, both with a high predictive and a non-predictive block. There were two main findings.

First, the results of the gaze-cueing experiment seem to suggest that the elevated psychopathy group (i.e., SRP-SF Total Score) is influenced differently by the predictivity of the blocks compared with the low psychopathy group. The results showed an overall slower response time in the high predictive blocks, compared to the non-predictive blocks for the low psychopathy group, a difference that was absent in the elevated psychopathy group. One explanation for this observation could be that participants used the cue more effectively because it was highly predictive (i.e. the high predictive blocks). This implies that valid cues guide attention more effectively, leading to a speeding up of reaction times, compared to valid trials in non-predictive blocks. Similarly, due to cues being more effective in the high predictive blocks, invalid cues in the high predictive blocks may show slowed reaction times compared to invalid cues in the non-predictive blocks. When the slowing on invalid trials is larger compared to the speeding up on valid trials, an overall slowing down can be observed in the high predictive blocks. This line of reasoning may explain why the low psychopathy group showed a reaction time difference between blocks whereas this was not the case for the high psychopathy group.

However, this finding should be interpreted with caution given that the initial three-way interaction between block predictivity, cue validity and SRP-SF Total Score did not reach significance. Also, this interaction was not observed in the arrow cueing experiment. Importantly, despite the group difference in response times for high and non-predictive blocks, individuals with elevated psychopathic traits did indeed become faster on valid trials in the high predictive block compared to those trials in the non-predictive block, mimicking the results of the low psychopathy group. Thus, weighing all relevant results it should be concluded that individuals with elevated psychopathic traits can pick up on secondary or contextual information (i.e., the predictivity of a cue).

This finding is contrary to our hypothesis and appears to be at odds with earlier findings by Hoppenbrouwers et al. (Hoppenbrouwers et al. 2015; Hoppenbrouwers et al. 2016b), who showed that elevated psychopathic traits are related to problems in integrating contextual information to guide top-down attention. A crucial difference however, is that in Hoppenbrouwers et al. (Hoppenbrouwers et al. 2016a; Hoppenbrouwers et al. 2015) the contextual information that could be used to guide top-down attention varied trial by trial, whereas contextual information (i.e., predictivity of the cue) was presented in a blocked manner in the current experiment. Therefore, when a certain probability (i.e., predictivity of a cue) is stable over a prolonged period of time, individuals with elevated psychopathic traits may learn how to use it.

**Fig. 2:**
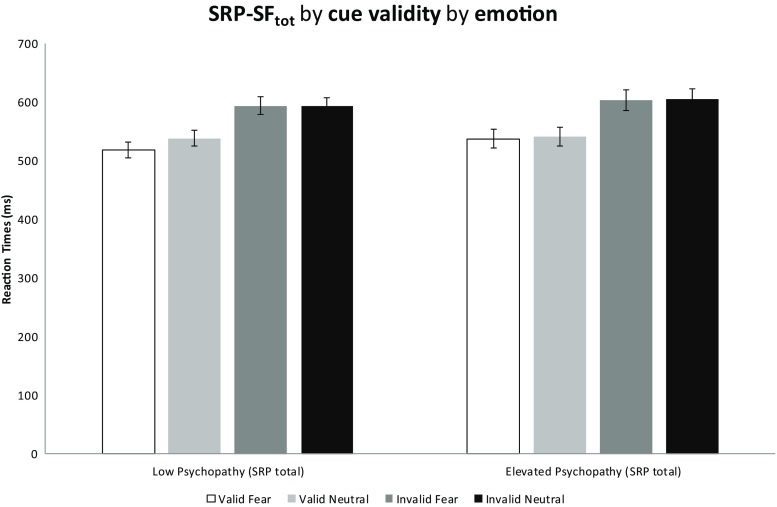
SRP total by cue validity by emotion interaction. This figure shows that the elevated psychopathy group uses the gaze-cue (as indicated by strong cueing effects) but no difference in the magnitude of the cueing effect is observed between fearful and neutral facial expressions. However, this difference in magnitude is observed in the low psychopathy group, as evidenced by significantly faster reaction times for valid fearful gaze-cues. Error bars denote standard error of the mean (SEM)

Second, we also observed that fearful facial expressions did not lead to faster reaction times in individuals with elevated psychopathic traits (see Fig. 2, and Table 2). Importantly, we did not observe any gaze-cueing deficits in relation to psychopathic traits. Rather, it appears that individuals with elevated psychopathic traits can use a gaze-cue adequately but that they are not affected by the fearful expression that is presented on the face. In other words, whereas a fearful face induced faster reaction times in the low psychopathy group this was not the case in the elevated psychopathy traits group. This suggests that in the low psychopathy group, fearful faces automatically induce a state of increased vigilance which in turn leads to faster deployment of attention. This finding is in line with a recent meta-analysis, (Hoppenbrouwers, Bulten, & Brazil, 2016) which showed that psychopathy is characterized by deficits in automatic threat detection and responsivity (Hoppenbrouwers et al. 2016b).

A few limitations should be noted. First, it is unlikely that the current sample would include individuals that would score over 30 on the PCL-R. Although our sample included 8 subjects with an SRP-SF Total score of over 70, indicating the presence of extreme psychopathic traits, replication of these findings in a large offender sample with a more objective PCL-R score would bolster the significance of these findings. In addition, the present sample size may be on the small side. With smaller samples, the chance of type 1 errors increases. Replication of the current findings in a larger community sample would therefore also increase confidence in the presently observed results. Nonetheless, our finding of reduced vigilance in response to a fearful face in individuals with elevated psychopathic traits does align with theoretical models of psychopathy (Blair 2013; Moul et al. 2012), which state that processing of fearful faces is impaired in psychopathy. As such, these findings add to a growing body of literature looking at the interaction between attention and emotion in psychopathy.

Second, connecting behavior on experimental tasks, such as the ones used here, with behavior in real-life situations is challenging. Typically, behavioral experiments report data that are in the range of milliseconds which makes it difficult to extrapolate experimental findings associated with psychopathy to actual psychopathic behavior. For example, the effect size for the low fear hypothesis was *r* = .21 (Hoppenbrouwers et al. 2016b), showing that deficient fear processing only explains ~4% of the variance in psychopathy. Important future lines along which research on psychopathy may develop are the integration of different models of psychopathy, together with more detailed computational models of attention and learning (see for instance (Brazil et al. 2017).

Taken together, our data suggest that individuals with elevated psychopathic traits can indeed pick up on secondary or contextual information when such information is stable over a prolonged period of time.
